# Screening for proline‑rich protein 11 gene expression in cervical cancer: Use as a novel diagnostic biomarker and poor prognostic factor

**DOI:** 10.3892/mi.2024.202

**Published:** 2024-11-11

**Authors:** Kaoutar Anouar Tadlaoui, Soukayna Alaoui Sosse, Ikram Tiabi, Mouna Aqerrout, Amal Souiri, Mustapha Benhessou, Moulay Mustapha Ennaji

**Affiliations:** 1 Research Team of Virology, Oncology and Biotechnologies, Laboratory of Virology, Oncology, Biosciences, Environment and New Energies, Faculty of Sciences and Techniques Mohammedia, University Hassan II of Casablanca, Mohammedia 28806, Morocco; 2 Research and Medical Analysis Laboratory of the Royal Gendarmerie Fraternity of Rabat, Rabat 10080, Morocco; 3 Faculty of Medicine and Pharmacy of Casablanca, University Hassan II of Casablanca, Casablanca 20250, Morocco

**Keywords:** proline-rich protein 11, cervical cancer, quantitative PCR, biomarker, diagnosis, prognosis

## Abstract

Cervical cancer is the fourth most common type of cancer affecting the female population worldwide, and it is associated with a late diagnosis and a poor prognosis. It is thus imperative to improve modern diagnostic methods by searching for novel tumor biomarkers, such as proline-rich protein 11 (PRR11), the expression of which is deregulated in various types of cancer and participates in their cellular progression. However, the involvement of PRR11 in cervical cancer has not yet been fully elucidated. The present study thus aimed to investigate the expression of PRR11 in cervical cancer as a diagnostic and prognostic biomarker, and to characterize the associated clinicopathological features. For this purpose, reverse transcription-quantitative PCR was used to assess the mRNA expression level of PRR11 in 100 cervical cancer and corresponding adjacent normal tissues. Statistical analysis was then performed to determine the association between PRR11 expression and the clinicopathological data of patients, and the overall survival rate of patients. The results revealed that PRR11 gene expression in cervical cancer tissues was significantly higher than that in corresponding normal adjacent tissues. No significant differences were found between PRR11 gene expression, age at diagnosis, FIGO stage, or histological type. Patients with a positive PRR11 expression had a shorter survival rate (P=0.035). As regards the diagnostic value, the results demonstrated that PRR11 expression was able to differentiate cancerous from normal cervical tissue with a sensitivity of 88.75%, a specificity of 100%, an area under the curve of 0.982 and a Youden index of 88.7%. Thus, PRR11 satisfies the standard criteria to be applied in diagnosis. On the whole, the present study demonstrates that PRR11 is well-expressed in cervical cancer tissues, is related to age and a poor prognosis, and may thus serve as a potential novel biomarker for cervical cancer diagnosis.

## Introduction

Among the female malignancies, cervical cancer (1) is the fourth type of cancer and the second leading cause of cancer-related mortality among the female population, with high incidence and mortality rates worldwide, with 604,127 (3.1%) new cases and 341,831 (4.1%) related deaths (1). In addition, CC is the leading cause of cancer-related death among females in 36 low- and middle-income countries (2). This is mainly due to late detection and poor prognoses, which reduces the chances of curative surgery.

The different types of cervical cancer are classified according to the tumor site and gene expression. A number of critical genes are linked to a higher incidence of the disease. Therefore, numerous genetic and epigenetic alterations that inactivate tumor suppressor genes and activate oncogenes play a major role in the pathogenesis of CC. Consequently, there is a need to explore novel and effective diagnostic biomarkers to better distinguish patients with CC in order to enable rapid diagnosis and thus the effective treatment of CC for improved and earlier cancer management.

Among the most potential biomarkers is proline-rich protein 11 (PRR11), a recently discovered gene in the amplification region of chromosome 17q22. Bioinformatics analysis has revealed that PRR11 comprises a bivalent nuclear localization signal, a pair of proline-rich regions and zinc finger domains, which are involved in the transduction of cell signals and mediate a cascade of cancer-related processes (3). Pertinent data have shown that PRR11 is a candidate oncogene in mammals, often playing a vital role in the initiation and progression, as well as other carcinogenic processes of various solid tumors, such as hilar cholangiocarcinoma (4), lung cance (5,6), pancreatic cancer (7), osteosarcoma (8), gastric cancer (9), breast cancer (10,11), colorectal cancer (12), ovarian cancer (13) and cervical cancer (14). In addition, PRR11 has been reported to commonly display extremely high expression levels in solid tumors and it is strongly related to local tumor recurrence and metastasis. However, although there is growing evidence that PRR11 is an influential tumor-related gene, the link between PRR11 and Moroccan women with CC remains questionable.

## Patients and methods

### Patients and specimens

A total of 100 fresh biopsies (80 tumor tissue samples and 20 corresponding adjacent normal tissues) were collected from patients with CC undergoing surgery following a histopathological examination at the Onco-Gynecology Department of the Mohammed IV Oncology Center in Casablanca, Morocco, between January, 2020 and December, 2021. Biopsies were sampled by physicians following standard protocols and immediately stored at -80˚C until analysis. Clinicopathologic data from enrolled cases were also collected according to the STROCSS guidelines (15). Patients who received chemotherapy and/or radiotherapy were excluded from the study.

The present study was ethically approved by the Biomedical Research Committee of the Faculty of Medicine and Pharmacy of Casablanca, Casablanca, Morocco (3/2018 on April 30, 2018). Free oral consent was obtained from all recruited patients and the confidentiality of their personal information was kept according to ethical rules.

### Total RNA extraction and cDNA synthesis

Total RNA was extracted from the tissue samples using TRIzol reagent® (Invitrogen; Thermo Fisher Scientific, Inc.) as instructed by the manufacturer. The NanoDrop 2000 spectrophotometer (Nanodrop Technologies, Inc.) was utilized to detect the concentration and purity of mRNA (considering the concentration of 1-2 µg). The High-capacity cDNA Synthesis Kit (Applied Biosystems; Thermo Fisher Scientific, Inc.) was used for reverse transcription according to the manual provided by the manufacturer.

### Quantitative qPCR (qPCR)

To assess the relative expression of the PRR11 gene, TaqMan^®^ Universal PCR Master Mix (2X) (Applied Biosystems; Thermo Fisher Scientific, Inc.) was used, as well as the TaqMan^®^ Gene Expression Assay (Applied Biosystems; Thermo Fisher Scientific, Inc.) which consists of a pair of unlabeled PCR primers and a specific TaqMan probe. Glyceraldehyde-3-phosphate dehydrogenase (GAPDH) was used as an internal endogenous control for normalizing gene expression. The primers used are listed in Table I. The thermocycling conditions involved an initial denaturation at 95˚C for 2 min, followed by 40 cycles of denaturation at 95˚C for 30 sec, annealing at 57˚C for 30 sec, and extension at 72˚C for 30 sec. The relative expression level of PRR11 was calculated using the 2^-ΔΔCq^ value, based on the threshold cycle (1) method (16).

### Statistical analysis

One-way ANOVA was conducted to assess the relative differential expression of PRR11 in CC tissues compared with adjacent normal tissues. The Chi-squared test was used examine the association between PRR11 expression levels and the clinicopathological features of the patients. Additionally, a ROC analysis was performed to evaluate the overall diagnostic performance of PPR11 as a biomarker in patients with CC. The Kaplan-Meier survival curve method was used analyze the overall survival probabilities of patients with CC according to the PRR11 expression level, with the Tarone-Ware test applied to analyze survival differences. Jamovi software, version 21.3.2 was used to perform all analyses, and a P-value <0.05 was considered to indicate a statistically significant difference; the confidence interval (CI) was 95%.

## Results

### Clinicopathological features of patients with CC

The clinicopathologic characteristics of the patients with CC included in the present study are summarized in Table II. The age at diagnosis of the patients with CC ranged from 27 to 85 years, with a mean age of 54 years (±10.99). The most common age group was ≥41 years, accounting for 81.25% of the cases. Clinical staging was performed according to the International Federation of Gynecology and Obstetrics (FIGO) classification and this revealed the predominance of stages I and II (91.25%). The predominant form of CC was squamous cell carcinoma, accounting for 82.5% of cases, while only 17.5% of the patients had adenocarcinoma.

### PRR11 mRNA expression in patients with CC

**Figure 1:**
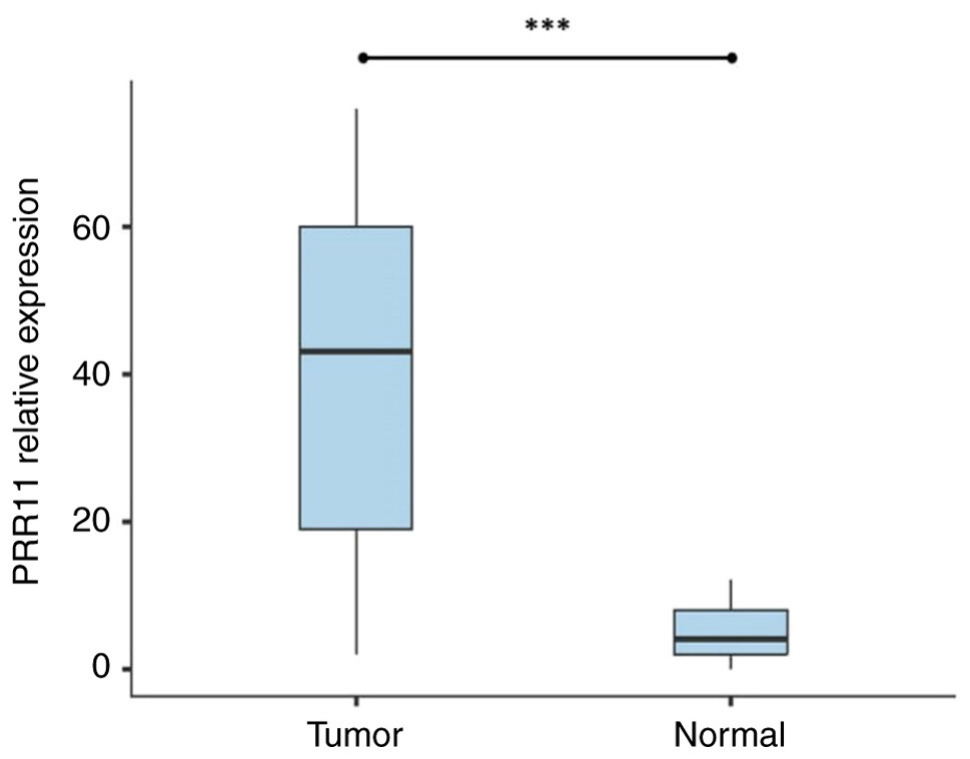
Relative expression levels of PRR11 mRNA in cervical cancer tissue compared with corresponding adjacent normal tissues. ^***^P<0.001. PRR11, proline-rich protein 11.

RT-qPCR was performed to assess the mRNA expression level of PRR11 in CC tumor tissues compared to corresponding adjacent normal tissues. The results revealed that PRR11 mRNA was expressed in 77.5% (62/80 cases) of the tumor tissues. It exhibited a relevant level of expression compared with the adjacent normal tissues; PRR11 mRNA expression was only found in 20% (4/20) of normal tissues. The relative expression of PRR11 was significantly higher in CC tissues than in adjacent normal tissues (P<0.001; Fig. 1).

### Association between PRR11 expression and clinicopathological characteristics of patients with CC

**Figure 2:**
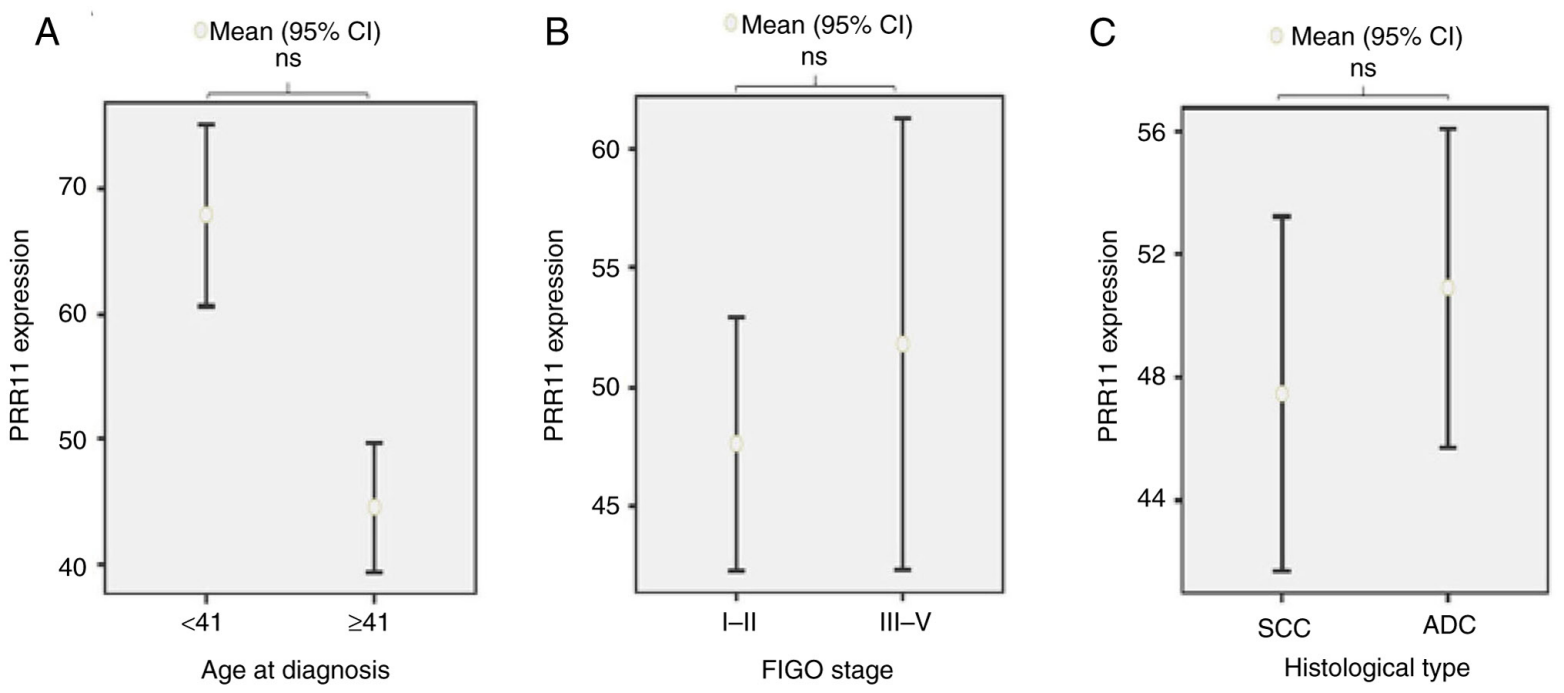
Association of PRR11 expression with some clinicopathological features. (A) Age at diagnosis, (B) FIGO stage, (C) histological type. SCC, squamous cell carcinoma; ADC, adenocarcinoma; ns, not significant; PRR11, proline-rich protein 11.

The results of the analysis of selected clinicopathological characteristics of patients with CC is presented in Table III and Fig. 2. No significant associations were observed between PRR11 expression in tumor tissues and clinicopathological characteristics, including age at diagnosis (P=0.308), FIGO stage (P=0.999), or histological type (P=0.506).

### PRR11 as a potential diagnostic biomarker for CC

**Figure 3:**
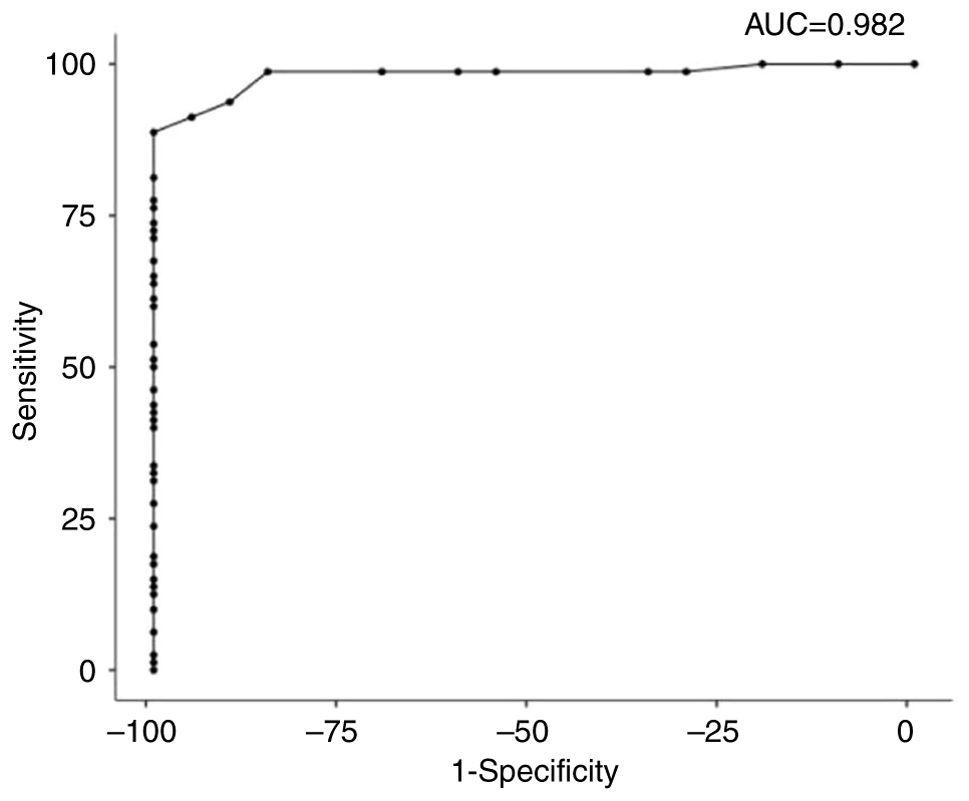
ROC analysis of PRR11 expression in cervical cancer tissues vs. normal adjacent tissues (AUC=0.982; sensitivity=88.75%; specificity=100%). PRR11, proline-rich protein 11.

ROC analysis was performed to examine PRR11 as a potential diagnostic biomarker for CC. The Roc analysis of CC vs. normal tissues revealed that PRR11 was a good potential diagnostic biomarker for discrimination between CC and non-tumor tissues. The sensitivity and specificity were 88.75 and 100%, respectively. The cut-off value was 13, the area under the ROC curve (AUC) was 0.982, and the P-value was <0.001. The positive predictive value (PPV) was 100%, which is the probability that the disease is present when the test is positive. The negative predictive value (NPV) was 68.97%, the probability that the disease is not present when the test is negative. The Youden index was 88.7%, which is >50%, thus indicating that the test satisfies the empirical criteria to be applied in the diagnosis of CC (Fig. 3 and Table IV).

### PRR11 overexpression related to the prognosis of CC

**Figure 4:**
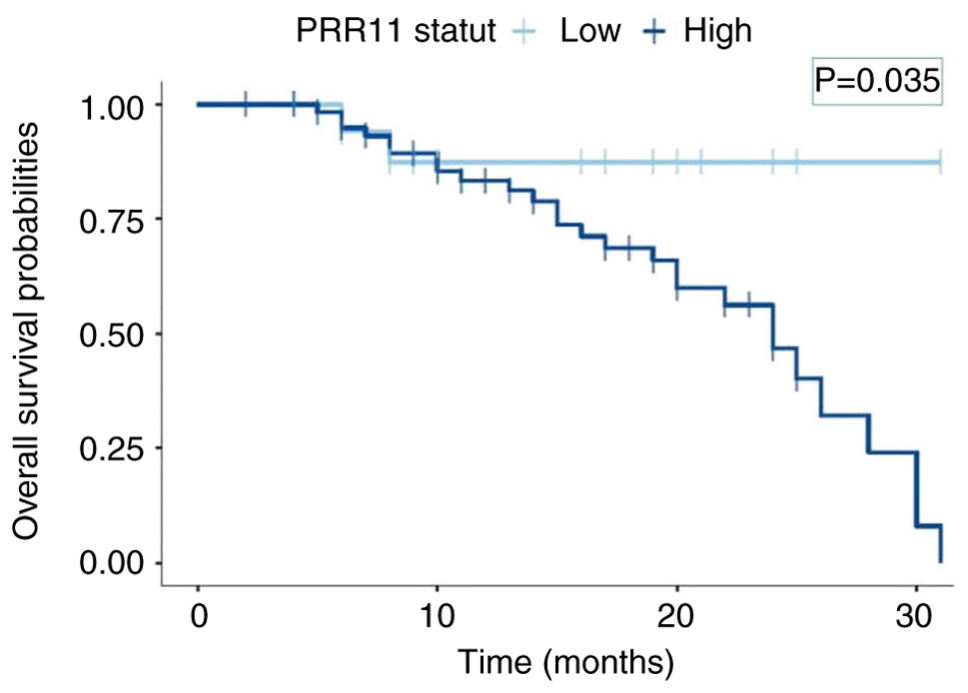
Kaplan-Meier analysis of overall survival for patients with cervical cancer according to the PRR11 expression level. PRR11, proline-rich protein 11.

To investigate the prognosis of PRR11 expression in CC, the Kaplan-Meier method was used to analyze the overall survival rate of 80 patients with CC followed-up for 36 months. A total of 30 patients succumbed and 50 patients survived. The median survival time of the 80 patients with CC was 27 months. Among the 62 patients with CC who were PRR11-positive, 27 succumbed and 35 survived. The median survival time for this event was 24 months. Of note, 2 patients out of 18 PRR11-negative patients with CC succumbed and 16 survived. The median survival time for PRR11-negative patients was 31 months. The survival time of patients with CC with a positive expression for PRR11 was significantly lower than that of patients with a negative expression of PRR11 (P=0.035; Fig. 4), suggesting that PRR11 may be a predictor of a poor prognosis in patients with CC.

## Discussion

CC is the fourth most common type of cancer affecting the female population. It is a well-known cause of mortality and is associated with a considerable socioeconomic burden worldwide (1). Multiple risk factors, ranging from genetic alterations to hormonal factors, environmental factors and viral etiology, are linked to the complex carcinogenesis of CC (1). The development of the majority of cases of CC is due to genetic mutations, common to most cancers, which result in either the overexpression of oncogenes or the inhibition of tumor suppressor genes (17). Therefore, to improve early detection and prevention of CC, there is a need to identify new and more reliable tumor biomarkers.

Over the past decade, researchers have reported that PRR11, a gene located in the 17q22 region of the chromosome, is a prominent candidate oncogene in mammals. PRR11 has been shown to be associated to several types of cancer, including ovarian cancer (13), gastric cancer (9), breast cancer (10,11), hilar cholangiocarcinoma (4), pancreatic cancer (7) and CC (14).

This has been well documented by the identification of PRR11 overexpression in these types of cancer and its obvious involvement in carcinogenesis and several other malignant biological processes of the cell cycle, such as cell proliferation, differentiation, migration, invasion, apoptosis, autophagy and cell resistance to chemotherapy (5,9,13,18-20).

The present study revealed that PRR11 mRNA was overexpressed in 77.5% of CC tissues vs. 20% of adjacent normal tissues, exhibiting a significantly higher level of expression in CC tissues compared with normal tissues (P<0.05). These findings are in concordance with those in the study by Xu and Chang (21), who first described that PRR11 was overexpressed in 76.67% of CC tissues compared with adjacent non-tumor tissues. Other studies have reported a significantly high expression of PRR11 in a number of types of cancer, such as lung cancer (5,6), ovarian cancer (13), carcinoma of the tongue (18), gastric cancer (9), breast cancer (10,11), hilar cholangiocarcinoma (4), colorectal cancer (12), pancreatic cancer (7) and osteosarcoma (8).

Based on the literature, PRR11 overexpression affects the cell cycle and promotes lung cancer progression (15), and the onset and development of CC (14,21). In addition, previous studies have indicated that PRR11 overexpression promotes ovarian cancer cell proliferation, migration and invasion by activating the PI3K/AKT/β-Catenin pathway (13) and promoting breast cancer cell progression and invasion by activating the epithelial-mesenchymal transition (EMT) process (10). Moreover, the functional study by Zhou *et al* (10) revealed that PRR11 decreased the expression of E-cadherin and cytokeratin-18, and increased the expression of vimentin, N-cadherin and fibronectin through EMT by targeting transcription factors [Slug, Snail, zinc finger E-box binding homeobox (ZEB)1 and ZEB2] (10,20).

However, it has been shown that the inactivation of PRR11 in CC cell lines increases the occurrence of apoptosis (21). PRR11 also has the potential to regulate apoptosis in CC cancer cells by stimulating the expression of caspase-3 proteins, the executive and irreversible factors in apoptosis (21), suggesting that PRR11 protein expression plays a critical oncogenic role in CC cell development and progression.

In the present study, PRR11 mRNA expression was not found to be significantly associated with any clinicopathological features of the patients, whereas opposite results were observed in the study by Zhao *et al* (14), which reported that PRR11 was associated with FIGO stage (P<0.05) in CC. In addition, Zhu *et al* (13) found that the overexpression of PRR11 in ovarian cancer tissues and cells was significantly associated with an advanced FIGO stage. Moreover, Xu and Chang (21) found that the expression level of PRR11 was significantly associated with the histological type of CC (P<0.05). By contrast, the difference was not statistically significant between CC and the patient age at diagnosis (P>0.05) (21), which is consistent with the results of the present study.

In addition, the results of the ROC analysis revealed that PRR11 represents a valuable biomarker for the early diagnosis of CC. This is consistent with previous studies reported in the literature, including research on lung cancer (5), pancreatic cancer (7), invasive breast cancer (11) and ovarian cancer (13).

On the other hand, the present study demonstrated that PRR11 has prognostic values and plays a role as a carcinogenic factor in CC. These findings are consistent with those of previous studies on PRR11, but in other types of cancer, such as breast cancer (10,11), hepatocellular carcinoma (22) and gastric cancer (23). Indeed, Wang *et al* (24) reported in their study that a high expression of PRR11 was a prognostic risk factor for patients with tongue squamous cell carcinoma, and this was also reported by Xu and Chang (21) in their study on CC.

Furthermore, there is evidence to indicate that PRR11 is a potential target for anticancer therapies in hilar cholangiocarcinoma and lung cancer (4,6). In this regard, it has been suggested that the regulation of PRR11 expression in CC cells can inhibit cell proliferation and promotes apoptosis through cyclin-D1 and caspase-3 proteins, rendering PRR11 a potential molecular target for CC treatment.

In conclusion, the present study confirmed PRR11 overexpression in CC tissues. Notably, PRR11 is emerging as a leading diagnostic biomarker candidate, and a factor for a poor prognosis. However, in order to promote the early identification and prevention of CC, the detailed underlying mechanisms of PRR11 in the cell cycle and carcinogenesis need to be further explored.

## Figures and Tables

**Table I:** Primer sequences of PRR11 and GAPDH targets.

Gene name	Gene symbol	Primer (5-3)
Proline-rich protein 11	PRR11	F: GACTTCCAAAGCTGTGCTTCC
		R: CTGCATGGGTCCATCCTTTTT
Glyceraldehyde 3-phosphate dehydrogenase	GAPDH	F: GGAGCGAGATCCCTCCAAAAT
		R: GGCTGTTGTCATACTTCTCATGG

**Table II:** Clinicopathological features of the patients with cervical cancer (n=80).

Features	No. of patients	Percentage
Age at diagnosis (years); mean age, 54 years		
<41	15	18.75
≥41	65	81.25
Histopathological grade		
I-II	73	91.25
III-IV	7	8.75
Histological type		
Squamous cell carcinoma	66	82.5
Adenocarcinoma	14	17.5

**Table III:** Association between PRR11 expression and some clinicopathological features of patients with cervical cancer.

		PRR11 positive expression		PRR11 negative expression		
Features	Effective	No. of patients	Percentage	No. of patients	Percentage	P-value
Age at diagnosis (years)						
<41	15	10	16.1	5	27.8	0.308
≥41	65	52	83.9	13	72.2	
FIGO stage						
I-II	73	56	90.3	17	94.4	0.999
III-V	7	6	9.7	1	5.6	
Histological type						
Squamous cell carcinoma	66	52	83.9	14	77.8	0.506
Adenocarcinoma	14	10	16.1	4	22.2	

PRR11, proline-rich protein 11.

**Table IV:** ROC curve analysis for PRR11 in patients with cervical cancer.

ROC curve data	Values
Cut-off value	13
Sensitivity (%)	88.75%
Specificity (%)	100%
Positive predictive value (PPV) (%)	100%
Negative predictive value (NPV) (%)	68.97%
Youden's index	0.887
Area under curve (AUC)	0.982
P-value	<0.001
No. of tumor tissue specimens	80
No. of control tissue specimens	20

PRR11, proline-rich protein 11.

## Data Availability

The datasets used and/or analyzed during the current study are available from the corresponding author on reasonable request.

## References

[1] Sung H, Ferlay J, Siegel RL, Laversanne M, Soerjomataram I, Jemal A, Bray F (2021). Global cancer statistics 2020: GLOBOCAN estimates of incidence and mortality worldwide for 36 cancers in 185 countries. CA Cancer J Clin.

[2] Bruni L, Serrano B, Roura E, Alemany L, Cowan M, Herrero R, Poljak M, Murillo R, Broutet N, Riley LM, de Sanjose S (2022). Cervical cancer screening programmes and age-specific coverage estimates for 202 countries and territories worldwide: A review and synthetic analysis. Lancet Glob Health.

[3] Ai Q, Bu YQ, Liu Z, Lan H, Ji Y, Du G, Yang ZM, Liu GL, Song FZ (2011). Structural and functional analysis of human PRR11 promoter. Chin J Biochem Mol Biol.

[4] Chen Y, Cha Z, Fang W, Qian B, Yu W, Li W, Yu G, Gao Y (2015). The prognostic potential and oncogenic effects of PRR11 expression in hilar cholangiocarcinoma. Oncotarget.

[5] Ji Y, Xie M, Lan H, Zhang Y, Long Y, Weng H, Li D, Cai W, Zhu H, Niu Y (2013). PRR11 is a novel gene implicated in cell cycle progression and lung cancer. Int J Biochem Cell Biol.

[6] Wang Y, Zhang Y, Zhang C, Weng H, Li Y, Cai W, Xie M, Long Y, Ai Q, Liu Z (2015). The gene pair PRR11 and SKA2 shares a NF-Y-regulated bidirectional promoter and contributes to lung cancer development. Biochim Biophys Acta.

[7] Tan S, Jiang Z, Hou A, Wang J, Zhang J, Dai L (2017). Expression of PRR11 protein and its correlation with pancreatic cancer and effect on survival. Oncol Lett.

[8] Li K, Yu H, Zhao C, Li J, Tan R, Chen L (2021). Down-regulation of PRR11 affects the proliferation, migration and invasion of osteosarcoma by inhibiting the Wnt/β-catenin pathway. J Cancer.

[9] Hu H, Song Z, Yao Q, Geng X, Jiang L, Guo C, Li H (2018). Proline-rich protein 11 regulates self-renewal and tumorigenicity of gastric cancer stem cells. Cell Physiol Biochem.

[10] Zhou F, Liu H, Zhang X, Shen Y, Zheng D, Zhang A, Lai Y, Li H (2014). Proline-rich protein 11 regulates epithelial-to-mesenchymal transition to promote breast cancer cell invasion. Int J Clin Exp Pathol.

[11] Anouar Tadlaoui K, Alaoui Sosse S, Benhessou M, El Karroumi M, Ennaji MM (2023). Proline-Rich Protein 11 Overexpression in Invasive Breast Carcinoma: A Potential Diagnosis Biomarker. Indian J Gynecol Oncolog.

[12] Zheng W, Zhu G, Huang Y, Hua J, Yang S, Zhuang J, Wang J, Huang Q, Xu J, Ye J (2017). PRR11 promotes growth and progress of colorectal cancer via epithelial-mesenchymal transition. Int J Clin Exp Med.

[13] Zhu J, Hu H, Wang J, Yang Y, Yi P (2018). PRR11 overexpression facilitates ovarian carcinoma cell proliferation, migration, and invasion through activation of the PI3K/AKT/β-catenin pathway. Cell Physiol Biochem.

[14] Zhao Z, Pang Z, Xu L, Chen Y, Yang Y (2020). Expression of PRR11 and SKA2 in cervical cancer tissues and its relationship with prognosis. Chin J Cancer Prev Control.

[15] Agha R, Abdall-Razak A, Crossley E, Dowlut N, Iosifidis C, Mathew G (2019). STROCSS 2019 Guideline: Strengthening the reporting of cohort studies in surgery. Int J Surg.

[16] Livak KJ, Schmittgen TD (2001). Analysis of relative gene expression data using real-time quantitative PCR and the 2(-Delta Delta C(T)) method. Methods.

[17] Lee EY, Muller WJ (2010). Oncogenes and tumor suppressor genes. Cold Spring Harb Perspect Biol.

[18] Wang C, Yu L, Ren X, Wu T, Chen X, Huang Y, Cheng B (2019). The oncogenic potential of PRR11 gene in tongue squamous cell carcinoma cells. J Cancer.

[19] Zhang L, Lei Y, Zhang Y, Li Y, Bu Y, Song F, Zhang C (2017). Silencing of PRR11 suppresses cell proliferation and induces autophagy in NSCLC cells. Genes Dis.

[20] Tadlaoui KA, Ennaji MM (2023). The molecular mechanism of novel oncogenes dysregulating signaling pathways associated with cervical carcinoma. Immunological Implications and Molecular Diagnostics of Genitourinary Cancer.

[21] Xu M, Chang L (2020). The Expression of PRR11 in Cervical Cancer and Its Effect on the Proliferation and Apoptosis of Cervical Cancer Cell. Labeling Immunoassays and Clinical Medicine.

[22] Qiao W, Wang H, Zhang X, Luo K (2019). Proline-rich protein 11 silencing inhibits hepatocellular carcinoma growth and epithelial-mesenchymal transition through β-catenin signaling. Gene.

[23] Song Z, Liu W, Xiao Y, Zhang M, Luo Y, Yuan W, Xu Y, Yu G, Hu Y (2015). PRR11 is a prognostic marker and potential oncogene in patients with gastric cancer. PLoS One.

[24] Wang C, Yu L, Hu F, Wang J, Chen X, Tai S, Cheng B (2017). Upregulation of proline rich 11 is an independent unfavorable prognostic factor for survival of tongue squamous cell carcinoma patients. Oncol Lett.

